# NucFuseRank: Dataset Fusion and Performance Ranking for Nuclei Instance Segmentation

**DOI:** 10.3390/bioengineering13080886

**Published:** 2026-07-31

**Authors:** Nima Torbati, Anastasia Meshcheryakova, Ramona Woitek, Sepideh Hatamikia, Diana Mechtcheriakova, Amirreza Mahbod

**Affiliations:** 1 Research Center for Medical Image Analysis and Artificial Intelligence, Department of Medicine, Faculty of Medicine and Dentistry, Danube Private University, 3500 Krems an der Donau, Austria; 2 Department of Pathophysiology and Allergy Research, Center of Pathophysiology, Infectiology and Immunology, Medical University of Vienna, 1090 Vienna, Austria; 3 Comprehensive Center for AI in Medicine (CAIM), Medical University of Vienna, 1090 Vienna, Austria; 4 Research Center for Medical Data Sciences and Artificial Intelligence (MeDSAI), Department of Medicine, Faculty of Medicine and Dentistry, Danube Private University, 3500 Krems an der Donau, Austria; 5 Austrian Center for Medical Innovation and Technology, 2700 Wiener Neustadt, Austria

**Keywords:** nuclei instance segmentation, dataset ranking, deep learning, computational pathology, H&E-stained images, medical image analysis

## Abstract

Nuclei instance segmentation in hematoxylin and eosin (H&E)-stained images plays an important role in automated histological image analysis, with various applications in downstream tasks. While several machine learning and deep learning approaches have been proposed for nuclei instance segmentation, most research in this field focuses on developing new segmentation algorithms and benchmarking them on a limited number of arbitrarily selected public datasets. In this work, rather than focusing on model development, we focused on the datasets used for this task. Based on an extensive literature review, we identified manually annotated, publicly available datasets of H&E-stained images for nuclei instance segmentation and standardized them into a unified input and annotation format. Using two state-of-the-art segmentation models, one based on convolutional neural networks (CNNs) and one based on a hybrid CNN and vision transformer architecture, we systematically evaluated and ranked these datasets based on their nuclei instance segmentation performance. Furthermore, we proposed a unified test set (NucFuse-test) for fair cross-dataset evaluation and a unified training set (NucFuse-train) for improved segmentation performance by merging images from multiple datasets. To the best of our knowledge, this is the first study to systematically benchmark and rank publicly available datasets for nuclei instance segmentation. By evaluating and ranking the datasets, performing comprehensive analyses, generating fused datasets, conducting external validation, and making our implementation publicly available, we provided a new protocol for training, testing, and evaluating nuclei instance segmentation models on H&E-stained histological images.

## 1. Introduction

Analyzing histological whole-slide images (WSIs) is considered the gold-standard method for the diagnosis and prognosis of various diseases, including different types of cancer [1]. To visualize cell and tissue structures, various staining types are commonly used. Among these, hematoxylin and eosin (H&E) staining is the most widely applied [2]. When particular protein markers need to be visualized, other staining techniques, such as immunohistochemistry, can also be applied [3].

Traditionally, histological image assessment has relied on conventional light microscopy, with pathologists examining tissue sections through microscope optics. By systematically navigating across regions of interest at multiple magnifications and applying their domain expertise, pathologists derive diagnoses [4]. With the advent of digital slide scanners, which enable scanning of histological slides into digital WSIs that can be stored on computers, numerous semi- and fully automatic approaches have emerged for histological image analysis [5], including tasks such as patch-based (in this study, the term patch refers to any image cropped from a larger image, such as a WSI) and whole-slide-based classification [6], segmentation of diverse anatomical parts of the tissues within the images [7,8], and histological image retrieval [9,10]. Among these tasks, nuclei instance segmentation is considered a fundamental step, as it provides relevant and essential information for downstream analysis tasks [11]. For instance, in breast cancer grading, nuclear density and nuclear counts are important features that can be derived when accurate nuclei instance segmentation masks are available [12,13].

**Figure 1:**
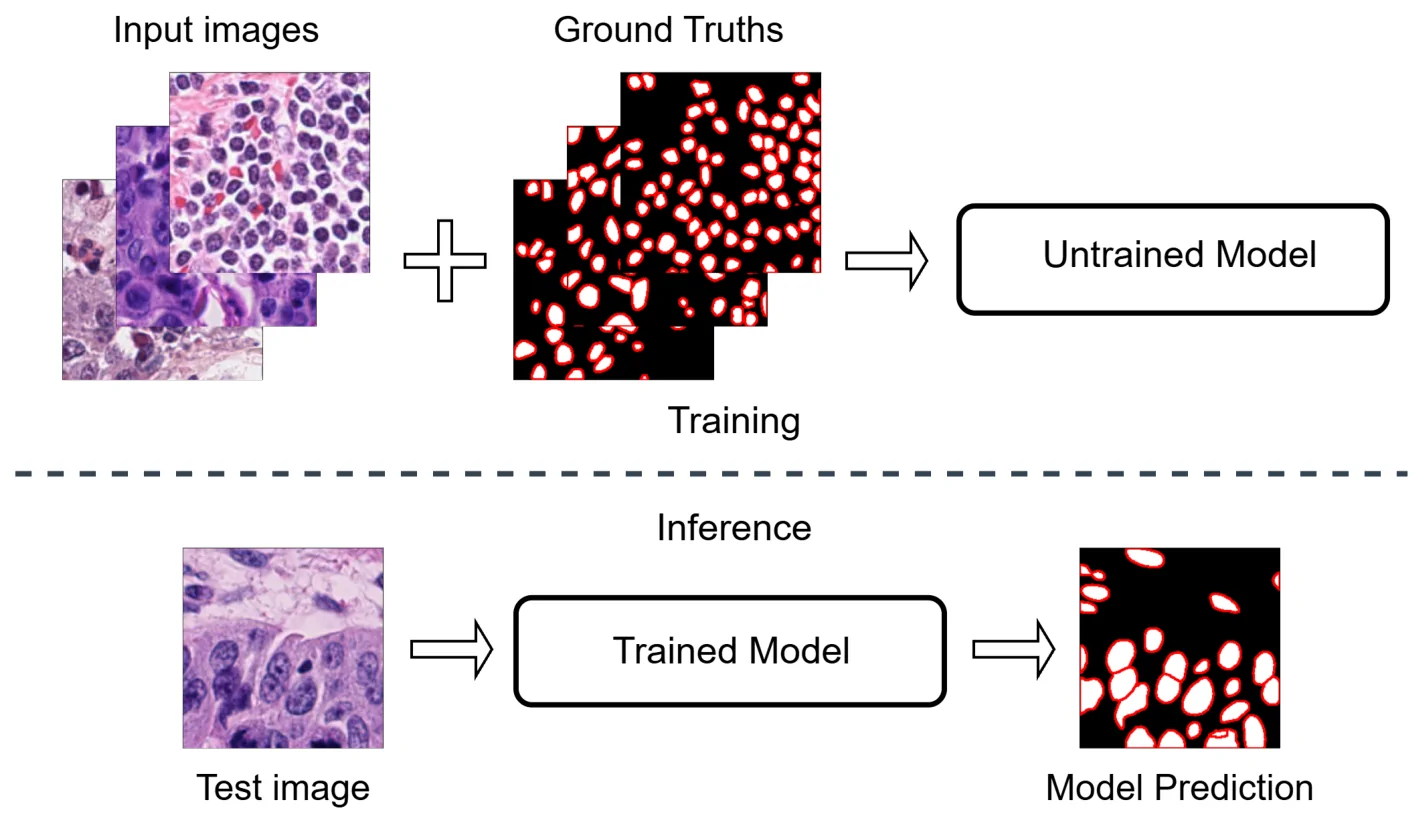
Overview of the conventional nuclei instance segmentation pipeline. During training (**top**), input images and their corresponding ground truth annotations are used to optimize the model parameters. These annotations, typically obtained through manual labeling, provide pixel-level delineation of individual nuclei. At inference (**bottom**), the trained model processes previously unseen images and produces predicted nuclei instance segmentation masks.

Regarding methodological approaches, various techniques and models have been proposed, ranging from conventional image processing methods to advanced machine learning- and deep learning (DL)-based approaches [14,15]. A typical pipeline for training and testing of supervised nuclei instance segmentation is shown in Figure 1. The pipeline consists of two main components: training and inference. During training, a model (e.g., a DL-based model) is trained using a set of input images with corresponding annotations. During inference, the trained model is applied to new unseen images with the aim of producing reliable and accurate segmentation predictions.

For nuclei instance segmentation, DL-based methods proposed in the literature can generally be categorized into two main groups [16,17]: (i) detection-based methods, such as Mask R-CNN and its enhanced variants [18,19], and (ii) segmentation-based approaches, including multi-encoder and multi-decoder convolutional neural network (CNN) architectures, as well as vision transformer (ViT)-based or hybrid CNN–ViT approaches. Models such as HoVerNet [20], HoVerNeXt [21], DDU-Net [17,22], Stardist [23] and CellViT [24] are examples of state-of-the-art DL-based approaches for nuclei instance segmentation and classification tasks. To evaluate these models, a number of publicly available datasets, such as MoNuSeg [11] or NuInsSeg [25] datasets, exist, which are commonly used as benchmarks.

Although many methods have been proposed for nuclei segmentation and detection, most research in this field has focused on developing or enhancing models, while comparatively little attention has been given to systematically evaluating the datasets used for training and benchmarking. These models are typically evaluated on a limited number of publicly available datasets, often selected arbitrarily from the larger pool of datasets available for nuclei instance segmentation. Moreover, the formats of input images and corresponding segmentation masks vary across datasets, which makes it difficult to seamlessly conduct straightforward and fair experimental comparisons when evaluating model performance. Since data quality plays a crucial role in the development of deep learning-based methods, dataset benchmarks are valuable resources for developing robust algorithms and providing insights into dataset characteristics [26]. In digital histopathology, recent benchmarking studies have primarily focused on evaluating the performance and generalizability of DL models including pathology foundation models [27], while relatively little research has been dedicated to dataset benchmarking. The lack of dataset-focused studies in this field, particularly for complex tasks such as nuclei instance segmentation, highlights the need for further research.

In this work, we focused on the datasets used for nuclei instance segmentation and aimed to provide a comprehensive benchmark and analysis of publicly available datasets used for model training and evaluation. To the best of our knowledge, this is the first study to systematically benchmark and rank publicly available datasets for this task. Specifically, our contributions are as follows:

1. We performed a thorough literature search to identify all manually annotated, publicly available datasets for nuclei instance segmentation and, based on predefined inclusion and exclusion criteria, selected 10 datasets that were subsequently standardized into a unified input image and segmentation mask format.
2. We introduced a unified test set (NucFuse-test) for fair cross-dataset evaluation by selecting a fixed number of images from each dataset based on predefined criteria.
3. Using two state-of-the-art segmentation models, one CNN-based and one hybrid CNN-ViT, we systematically evaluated and ranked the datasets based on their generalization performance on NucFuse-test.
4. We constructed a unified training set (NucFuse-train) by merging training images from multiple datasets and investigated its effect on segmentation performance.
5. We made all code and all created datasets, including NucFuse-test and NucFuse-train, publicly available for future research and benchmarking.

## 2. Methods

**Figure 2:**
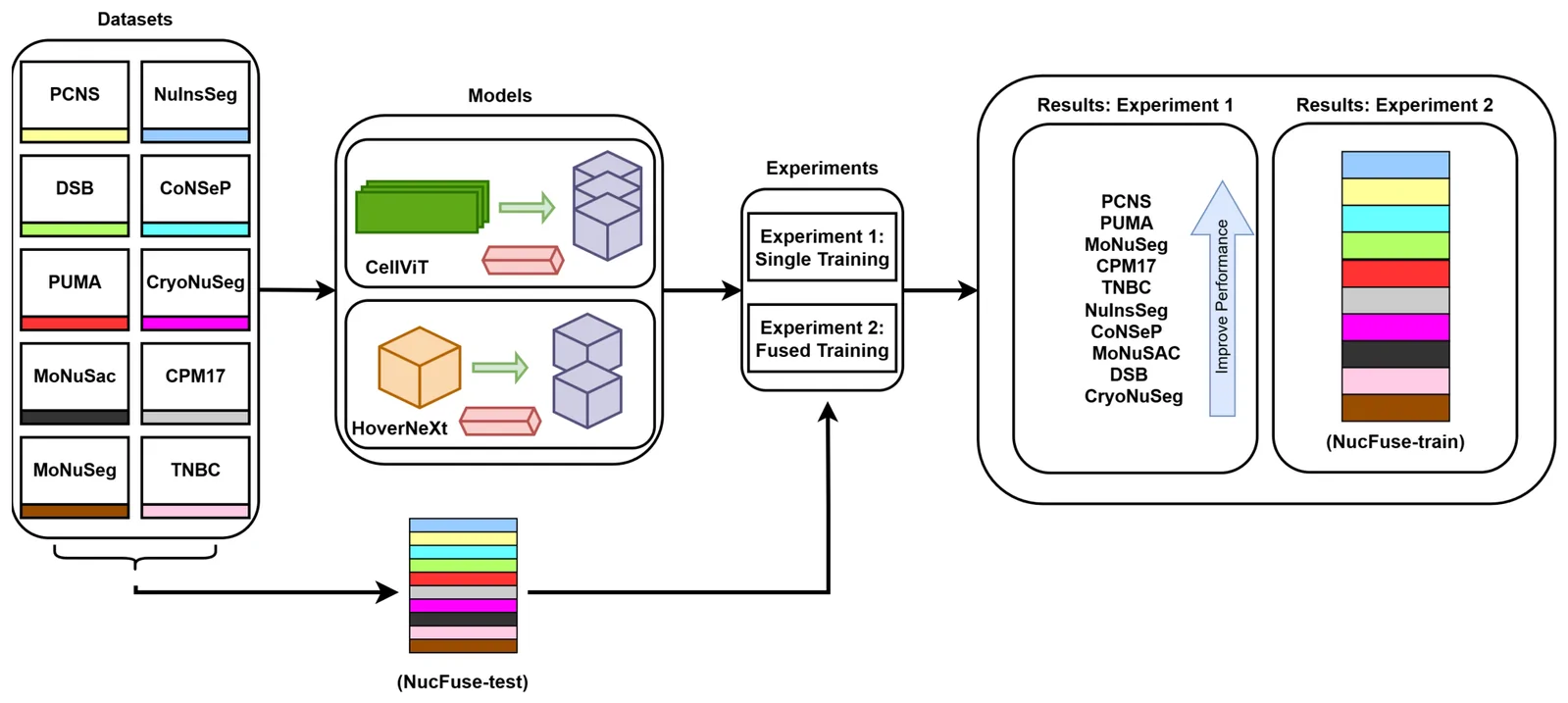
An overview of the entire workflow. First, a unified test set (NucFuse-test) was constructed from all datasets to evaluate the experiments. The remaining images were then used to train two state-of-the-art models, CellViT [24] and HoVerNeXt [21], under two experimental setups: single-dataset training (e.g., training only on PCNS or only on NuInsSeg) and fused-dataset training (progressively merging the training datasets). Based on the results of Experiment 1, the datasets were ranked according to their performance on the unified test set. Based on the results of Experiment 2, an optimal merged training dataset (NucFuse-train) was proposed.

An overview of the entire pipeline is shown in Figure 2. The overall goal was to analyze and rank publicly available datasets for the nuclei instance segmentation task based on the performance of models trained on them and evaluated on a unified test set and to introduce a new merged training dataset. The results of this study can serve as a benchmark for dataset selection for model training and for model evaluation in future research.

To achieve these goals, we first constructed a unified test set (NucFuse-test) by selecting a fixed number of images from each dataset based on predefined criteria. The remaining images were then used to train two state-of-the-art models, CellViT [24] and HoVerNeXt [21], under two experimental setups: single-dataset training and fused-dataset training. The experimental results from single-dataset training were used to rank the datasets, and results from fused-dataset training were used to form an optimal merged training dataset (NucFuse-train).

In the following subsection, the details of our approach are described.

### 2.1. Datasets

In this work, we compiled publicly available, manually annotated datasets for the task of nuclei instance segmentation. In addition to datasets originally designed for this task, such as MoNuSeg [11] and CryoNuSeg [28], we also included nuclei instance segmentation and classification datasets, such as MoNuSAC [29] and CoNSeP [20]. For the latter datasets, all classes were merged into a single foreground class to make them suitable for the nuclei instance segmentation task. This results in a total of 10 datasets, of which seven were originally designed for nuclei instance segmentation, and three were derived from nuclei instance segmentation and classification datasets. The main statistics of these datasets are summarized in Table 1 (No. 1 to No. 10). For each dataset, Table 1 reports the total number of image patches, the total number of annotated nuclei, the number of organs from which the images of tissue sections were obtained, and the patch size (if multiple sizes are present, the range of patch sizes). Additionally, for datasets that include nuclei classes, this information is indicated in the classification column of the table. Furthermore, the table includes the nuclei density, defined as the mean number of annotated nuclei per patches with 256×256 pixels for each dataset. A brief description of each dataset is provided in the following subsections.

#### 2.1.1. PCNS

The Pan-Cancer Nuclei Segmentation (PCNS) dataset, introduced by Hou et al. [30] in 2019, is one of the largest publicly available histopathology nuclei segmentation datasets. It contains 1356 image patches with 37,313 manually annotated nuclei collected from tissue specimens from nine different organs.

#### 2.1.2. PUMA

The Panoptic Segmentation of Nuclei and Tissue in Advanced Melanoma (PUMA) dataset, introduced by Schuiveling et al. [31] in 2025, was released as part of the PUMA challenge for nuclei instance segmentation and classification. It consists of 205 image patches with 97,187 annotated nuclei from tissue specimens from eight organs. In addition to nuclei instance annotations, the dataset provides nuclei class labels and tissue annotations, enabling the evaluation of both segmentation and classification performance.

#### 2.1.3. MoNuSeg

The MoNuSeg dataset, introduced by Kumar et al. [11] as part of the MICCAI 2018 Multi-Organ Nuclei Segmentation Challenge, has become one of the most widely used benchmarks for nuclei instance segmentation. It contains 51 H&E-stained image patches with 30,827 manually annotated nuclei collected from tissue specimens from nine organs.

#### 2.1.4. CPM17

The CPM17 dataset was introduced by Vu Dang et al. [32] for the MICCAI 2017 Computational Precision Medicine Challenge. It contains 64 image patches with 7570 annotated nuclei collected from four cancer types (non-small-cell lung cancer, head and neck squamous-cell carcinoma, glioblastoma multiforme, and low-grade glioma), corresponding to three anatomical sites.

#### 2.1.5. TNBC

The TNBC dataset, introduced by Naylor et al. [33] in 2018, consists of histopathology images from patients with triple-negative breast cancer. It contains 50 image patches with 4056 manually annotated nuclei from breast tissue.

#### 2.1.6. NuInsSeg

The Nuclei Instance Segmentation (NuInsSeg) dataset, introduced by Mahbod et al. [25] in 2024, is a large multi-organ nuclei instance segmentation dataset containing 665 image patches with 30,698 manually annotated nuclei collected from tissue specimens from 31 organs.

#### 2.1.7. CoNSeP

The Colorectal Nuclear Segmentation and Phenotypes (CoNSeP) dataset, introduced by Graham et al. [20] in 2019, was developed for simultaneous nuclei segmentation and classification in colorectal tissue. It contains 41 image patches with 24,332 manually annotated nuclei from colorectal specimens.

#### 2.1.8. MoNuSAC

The MoNuSAC dataset, introduced by Verma et al. [29], extends the MoNuSeg challenge by incorporating nuclei classification in addition to instance segmentation. It contains 310 image patches with 46,828 manually annotated nuclei collected from tissue specimens from four organs. The dataset includes images of varying sizes and provides instance-level class annotations for multiple nuclei categories.

#### 2.1.9. DSB

The Data Science Bowl (DSB) 2018 dataset, introduced by Caicedo et al. [34], was released as part of the Kaggle Data Science Bowl competition on nuclei segmentation. It contains 107 microscopy image patches with 4529 manually annotated nuclei.

#### 2.1.10. CryoNuSeg

The CryoNuSeg dataset, introduced by Mahbod et al. [28] in 2021, contains cryosectioned H&E-stained histopathology images acquired from tissue specimens from 10 organs. It consists of 30 image patches with 7592 manually annotated nuclei. Unlike most datasets based on formalin-fixed paraffin-embedded (FFPE) tissue, CryoNuSeg focuses on frozen sections, providing a benchmark for evaluating model robustness across different tissue preparation protocols.

#### 2.1.11. Excluded Datasets

These 10 selected datasets were chosen from a list of 24 publicly available datasets for nuclei instance segmentation or detection in histology images. The complete list of excluded datasets is reported in Table S1 of the Supplementary Materials, together with the corresponding main reason for exclusion.

Since our focus was on the instance segmentation task, we excluded datasets designed only for detection, such as OCELOT [36], as these datasets provide annotations only for nuclei centers. Additionally, we excluded datasets that are subsets of larger datasets; Kumar [37] and CPM-15 [32] fall into this category. We also excluded weakly annotated datasets, such as Janowczyk [38]. In these datasets, either only parts of the images are annotated or only specific nuclei classes are annotated. Furthermore, we excluded datasets such as NuCLS [39] and Lizard [40], which rely on semi-automatic annotations. We did not include these datasets mainly for two reasons. First, semi-automatic pipelines typically employ a DL backbone to generate initial annotations, which may introduce implicit bias toward the characteristics of the model used for annotation. Second, the backbone models are often pretrained using other publicly available datasets; therefore, including such semi-automatically annotated datasets in our experimental setup could introduce unintended dependencies and potential data leakage, compromising the fairness of the evaluation. However, among the semi-automatically annotated datasets, we retained PanNuke [35] dataset (11th dataset in Table 1) for comparison and external validation purposes. Finally, we excluded datasets such as UCSB [41], for which either the input images or the corresponding segmentation masks are no longer available.

### 2.2. Merged Test and Train Sets

To validate the performance of the studied models, we constructed a unified test set (NucFuse-test) using samples from all datasets considered in this study. Specifically, 14 image patches were selected from each dataset. If a dataset originally included a test set or a validation set, the 14 image patches were selected from these subsets, with priority given to the test set. If a dataset did not provide either a test or a validation set, the 14 image patches were randomly sampled from the entire dataset. We chose the value of 14 because MoNuSeg [11] has the smallest test set among the datasets with an official test split, containing 14 images. We also set a minimum patch-size threshold of 256×256 pixels when sampling the test set. For the MoNuSAC dataset, images containing macrophages were excluded to reduce bias toward this dataset, as macrophages were only included in MoNuSAC, and their annotations/segmentations were generally less reliable [42,43]. The resulting NucFuse-test set reduces the influence of datasets with larger sample sizes while preserving the original structure of each dataset. After selecting 14 image patches per dataset, all remaining image patches were used for training. By progressively merging training image patches across datasets, we also created a merged training dataset (NucFuse-train) to investigate whether combining publicly available data could improve nuclei instance segmentation performance.

We make all generated datasets publicly available on Kaggle at https://www.kaggle.com/datasets/nimatorbati/nucfuserank-nuclei-instance-segmentation/data (accessed on 28 July 2026). The repository contains four main folders. The first folder, named original_split, contains all datasets in their original form (original train/test split, or the entire dataset if no official split exists), using an identical input image format (.tif) and segmentation mask format (.npy). The second folder, named custom_split, contains all datasets using the input image and segmentation mask formats based on our custom split. The third folder, named merged_split, contains NucFuse-test and NucFuse-train. Finally, to enable a compact overview and visualization, we also provide a separate folder containing only the images from NucFuse-test.

### 2.3. Models

To evaluate and compare instance segmentation performance across the datasets, we employed two state-of-the-art segmentation models: HoVerNeXt and CellViT. HoVerNeXt is among the latest CNN-based architectures, while CellViT belongs to the ViT family. Both models are originally capable of performing nuclei classification; however, this functionality was disregarded in our experiments, as we focused solely on the nuclei instance segmentation task in this study.

#### 2.3.1. HoVerNeXt

In an encoder–decoder architecture, HoVerNeXt integrates ConvNeXt-V2 [44] encoders to enhance nuclei segmentation accuracy in histological images. Its decoder consists of two parallel branches: a class decoder and an instance decoder. In our experiments, only the instance decoder was used. HoVerNeXt is also much faster than its predecessor, HoVerNet [20], while providing comparable results.

#### 2.3.2. CellViT

CellViT is an encoder–decoder-based model featuring a single encoder and three decoder branches. The novelty of CellViT lies in its use of a ViT [45] as the encoder backbone. To achieve optimal performance, the model leverages ViT encoders pretrained on histological image data. Similar to HoVerNet, the decoder consists of three branches responsible for binary segmentation, class prediction (disregarded in our experiments), and distance map estimation. Equipped with its ViT encoder, CellViT achieved superior performance compared to many other models, including HoVerNet.

## 3. Experiments

This section presents the experimental setup and evaluation details. We conducted two main experiments, along with two additional complementary experiments, as described below.

### 3.1. Experiment 1: Single-Dataset Training

In this set of experiments, we trained independent HoVerNeXt and CellViT models using the training data of each individual dataset listed in Table 1 (No. 1 to No. 10). To ensure a uniform training scheme, we first generated non-overlapping 256×256 pixel patches from all images in each dataset. If the size of an image was smaller than 256 pixels in either dimension, we applied white-padding to increase its size to 256×256 pixels. The number of patches with 256×256 pixels used for training each dataset is reported in the last column of Table 1. We used an 80%/20% split for training and validation, respectively. All trained models were evaluated on the generated unified test set (NucFuse-test) and were subsequently ranked based on their nuclei instance segmentation performance.

### 3.2. Experiment 2: K-Best Dataset Training

Using the rankings obtained from Experiment set 1, we constructed fused datasets by combining the top-*k* best datasets to identify the optimal joint training set. For this purpose, we trained both models (HoVerNeXt and CellViT) on fused datasets for k=1 to k=10, where k=9 represents the NucFuse-train dataset. The rationale for choosing k=9 for NucFuse-train is provided in Section 4.2. Similar to Experiment set 1, we evaluated the performance of all models using the unified test set (NucFuse-test).

### 3.3. Additional Experiments

While the main focus of this study is on Experiment 1 and Experiment 2, we also performed two additional complementary experiments. These experiments investigated (1) the effect of stain normalization on performance, and (2) compared results with a semi-automatically generated dataset (PanNuke [35]) and with a recently developed foundation model for nuclei and cell segmentation (CellSAM [46]).

#### 3.3.1. Effect of Stain Normalization

Within an additional experiment, we investigated the effect of stain normalization on model performance. Stain normalization is a widely used preprocessing technique in computational pathology that aims to reduce color variability caused by differences in tissue preparation, staining protocols, scanner characteristics, and laboratory-specific procedures. Such variability can introduce unwanted domain shifts that can negatively affect the generalization of DL models [47]. We investigated the effect of stain normalization using multi-target stain normalization (Avg-post technique) [48] and the non-deterministic stain augmentation proposed in [49]. We selected seven images from tissue specimens from seven different organs in the MoNuSeg dataset as reference images for the non-deterministic stain augmentation during training, which was applied with a probability of 50% [49]. To conduct these experiments, a setup similar to Experiment 1 was applied, where models were trained on a single dataset and evaluated on the entire NucFuse-test set.

#### 3.3.2. PanNuke and CellSAM

While this study focuses on publicly available, manually annotated datasets for nuclei instance segmentation, we also performed additional comparative experiments using models trained on the PanNuke dataset [35], a semi-automatically generated dataset, and CellSAM [46], a recently developed general-purpose foundation model for cell and nuclei segmentation. For the PanNuke experiment, we trained HoVerNeXt and CellViT using the PanNuke training data and evaluated their performance on the NucFuse-test set. We also evaluated the performance of the CellSAM model on NucFuse-test.

### 3.4. Evaluation

For evaluation, we employed widely used metrics including precision, recall, and panoptic quality (PQ). Among these metrics, PQ is the most commonly used index for benchmarking [17,20,29]; therefore, we used it as the main evaluation metric for dataset ranking in our experiments. The details of these indices are described below:

- Precision:
  $$\mathrm{Precision}=\frac{TP}{TP+FP}$$
- Recall:
  $$\mathrm{Recall}=\frac{TP}{TP+FN}$$
- Panoptic quality (PQ):
  $$\mathrm{PQ}=\frac{\sum_{(p,g)\in TP}\mathrm{IoU}\left(p,g\right)}{\left|TP\right|+\frac{1}{2}\left|FP\right|+\frac{1}{2}\left|FN\right|}$$
  where TP denotes predicted instances that can be uniquely paired with a ground truth instance with IoU>0.5; instances that cannot be paired are counted as FP. FN represents ground truth instances that are not paired with any prediction. IoU is the intersection over union of a paired ground truth and predicted instance.

For a better comparison between datasets, we also defined a cross-dataset score matrix as follows:

$$C_{i,j}=\frac{\mathrm{PQ}\left(D_{i},D_{j}\right)+\mathrm{PQ}\left(D_{j},D_{j}\right)}{2\;\mathrm{PQ}(D_{j},D_{j})}$$

where $C_{i,j}$ denotes the cross score between datasets $D_{i}$ and $D_{j}$, and $\mathrm{PQ}(D_{i},D_{j})$ represents the PQ on the test set derived from dataset $D_{j}$ when the model is trained on dataset $D_{i}$.

For dataset ranking and comparison, we also report 95% confidence intervals obtained using nonparametric bootstrapping over the 140 test images, as well as *p*-values for selected comparisons based on paired *t*-tests [50,51].

### 3.5. Implementation Details

All experiments were conducted on a workstation equipped with an AMD Ryzen 9 7950X CPU, 96 GB of RAM, and an NVIDIA RTX 4090 GPU. For both models, we adhered to the original implementations for training and inference. Specifically, we used convnextv2tinyencoder for HoVerNeXt and the ViT256 encoder for CellViT. All models were trained with a batch size of 5 for 300 epochs. Since both models output horizontal and vertical distance maps, these maps were used in the post-processing step for nuclei instance segmentation. Our full training and evaluation implementation is available in our GitHub repository: https://github.com/masih4/NucleiAnalysis (accessed on 28 July 2026).

## 4. Results & Discussion

The experimental results and discussion from Experiment 1, Experiment 2, and additional experiments are presented in this section.

### 4.1. Experiment 1 Results: Single-Dataset Training

The results of Experiment 1, described in Section 3.1, in terms of precision, recall and PQ, are shown in Table 2. Results are reported for both HoVerNeXt and CellViT. The datasets were ordered and ranked based on their mean PQ scores on the NucFuse-test set. While the last column shows the exact ranking of the datasets, we also categorized them into four groups (separated by dashed lines in the table). PCNS, PUMA, and MoNuSeg form the first group and can be considered the three best-performing datasets for both HoVerNeXt and CellViT, with a large margin compared to the other datasets. Among the first group, for HoVerNeXt, the paired *t*-test showed no significant difference between PUMA and PCNS (*p* = 0.446). However, PUMA significantly outperformed MoNuSeg (*p* = 0.008), and the difference between PCNS and MoNuSeg was also significant (*p* = 0.042). For CellViT, paired *t*-tests showed no significant differences between any of the top three datasets (*p* > 0.05). CPM17 is in the second group; although inferior to the datasets in the first group, it still delivers substantially better average performance than the lower-ranked datasets. This observation was also confirmed with the paired *t*-test, where MoNuSeg (the lowest-performing dataset in the first group) showed statistically significant superior performance compared to CPM17 (*p* = 0.003 for HoVerNeXt and *p* < 0.0001 for CellViT). TNBC, NuInsSeg, CoNSeP, and MoNuSAC are in the third group. Statistical analysis showed that CPM17 significantly outperformed TNBC (the best-performing dataset in the third group) with *p* < 0.0001 for both models. Finally, DSB and CryoNuSeg are in the fourth group, showing the lowest average performance. Statistical comparison between DSB (the best-performing dataset in the fourth group) and MoNuSAC (the lowest-performing dataset in the third group) showed statistically significant superior performance of MoNuSAC for both models (*p* < 0.0001 for HoVerNeXt and *p* = 0.037 for CellViT).

Of note, the WSIs used to create the CryoNuSeg dataset are based on frozen-sectioned tissue samples, which, due to different preparation techniques, may result in different image quality. This may limit generalization and lead to inferior performance compared to other datasets, which are mainly based on standard formalin-fixed paraffin-embedded (FFPE) samples [28].

Furthermore, as the ranking indicates, some datasets with smaller training sample sizes can still deliver better performance than datasets with larger training sets. For example, CPM17 and TNBC, with only 285 and 144 training samples, respectively, achieved better overall results compared to much larger datasets such as NuInsSeg and MoNuSAC, which contain 2604 and 2692 training samples, respectively. This finding confirms that training set size is not the only contributing factor to improved performance, and that other criteria, such as annotation quality and diversity in the training images, influence the results.

Comparing the models, HoVerNeXt and CellViT achieved comparable performance, with mean PQ scores of 48.10% for HoVerNeXt and 47.84% for CellViT, with HoVerNeXt performing slightly better. CellViT’s performance could likely be improved using a larger encoder (e.g., incorporating a pretrained foundation model as the encoder, similar to CellViT++ [52]); however, achieving the highest possible performance was not the primary objective of this study. In terms of model size, CellViT is larger than HoVerNeXt, with 46.7 million trainable parameters compared with 36.1 million trainable parameters for HoVerNeXt. In terms of computational cost, for a dummy RGB input image of size 256×256 pixels, CellViT requires approximately 217.2 GFLOPs, whereas HoVerNeXt requires approximately 24.1 GFLOPs. Thus, CellViT has about 1.3 times more parameters and requires roughly 9 times more floating-point operations than HoVerNeXt. Therefore, HoVerNeXt may be preferable when computational resources are limited.

It is also worth notifying that for the higher-ranked datasets, particularly those in group 1 and group 2, the performance differences between HoVerNeXt and CellViT were smaller than for the lower-ranked datasets. For instance, for the PCNS and PUMA datasets, the PQ differences between the two models were 0.42% and 1.01%, respectively, whereas for DSB and CryoNuSeg, the performance differences were much larger (5.39% and 5.25%, respectively).

**Figure 3:**
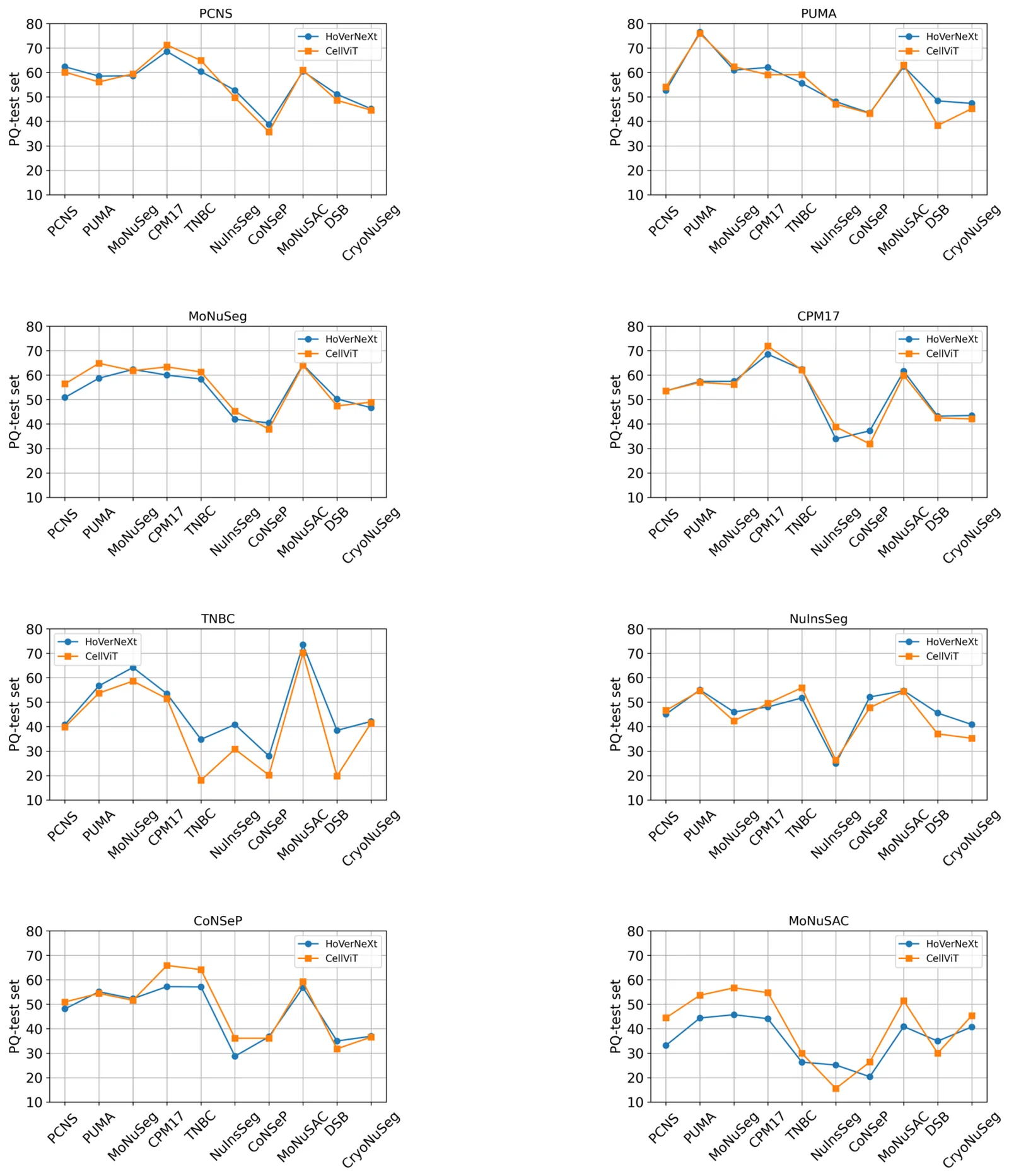

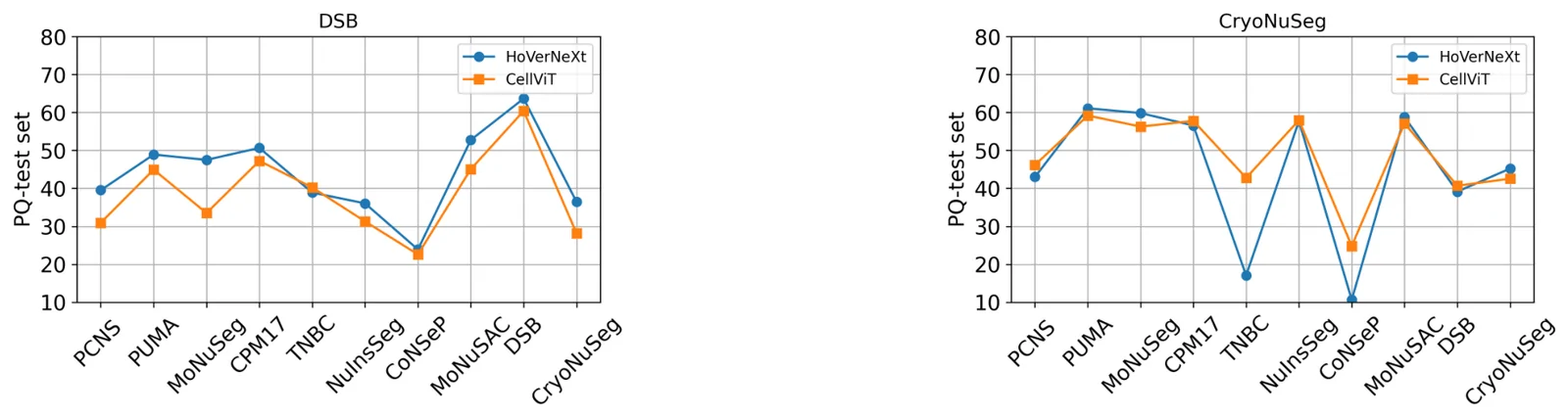
Results from single-dataset training. For each training dataset, HoVerNeXt and CellViT were trained using only the corresponding dataset, and their instance segmentation performance based on the panoptic quality (PQ) score was evaluated separately on each constituent subset of the unified test set, NucFuse-test.

While Table 2 reports the performance of HoVerNeXt and CellViT for each individual dataset evaluated on the entire NucFuse-test set, we provide a detailed breakdown analysis in Figure 3. This figure consists of ten subfigures, each showing performance across the different constituent subsets of the NucFuse-test set. For example, in the first subfigure (PCNS), both CellViT and HoVerNeXt are trained on the PCNS training data, and their performance is reported separately for each subset of NucFuse-test (test samples from PCNS, PUMA, MoNuSeg and others). The average performance for each test subset is reported in Table S2 in the Supplementary Materials, where overall, the models achieve the best performance on the CPM17 test subset and the worst performance on the CoNSeP test subset.

**Figure 4:**
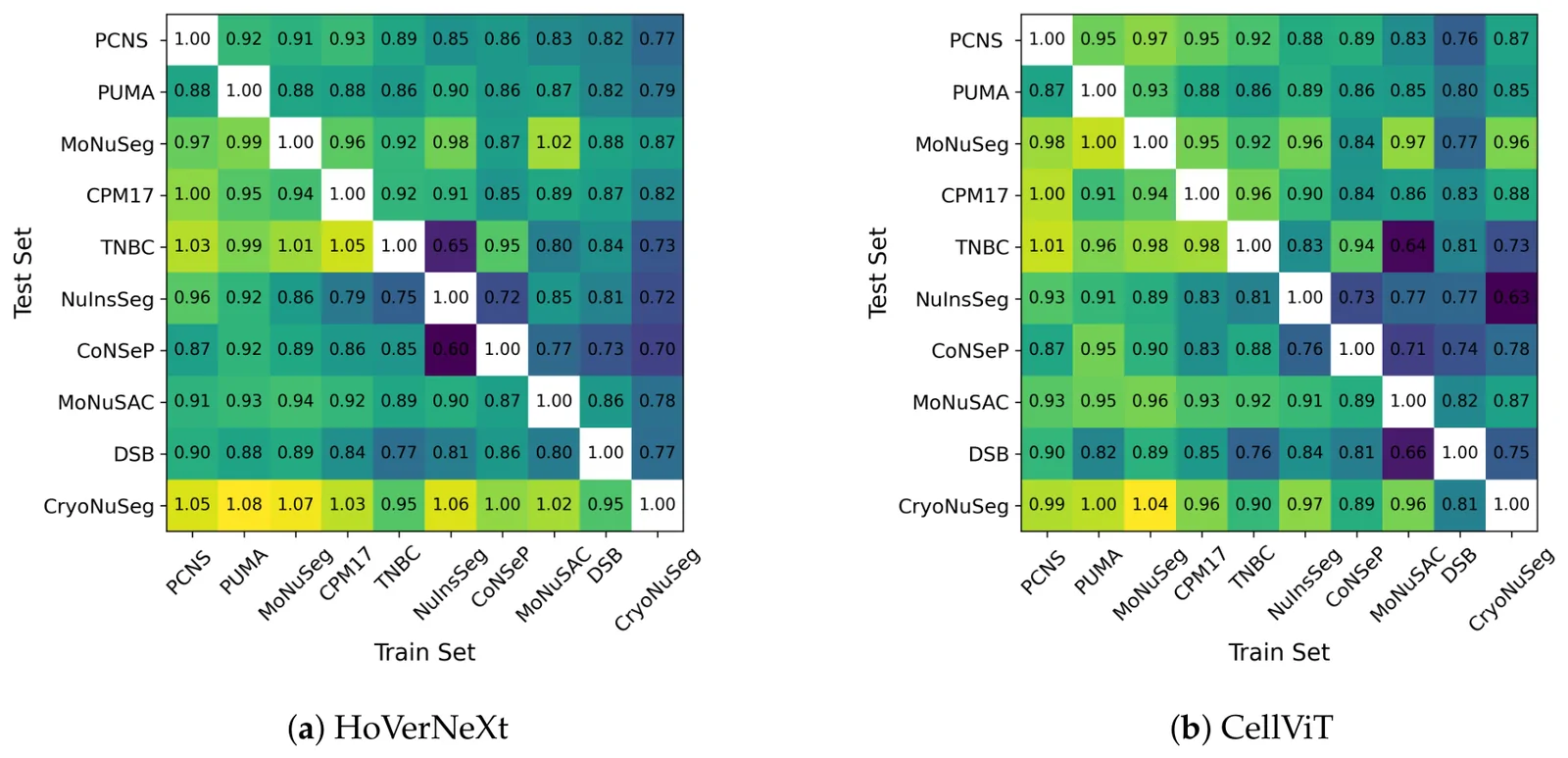
Cross-dataset score matrices for HoVerNeXt (**a**) and CellViT (**b**) models.

**Figure 5:**
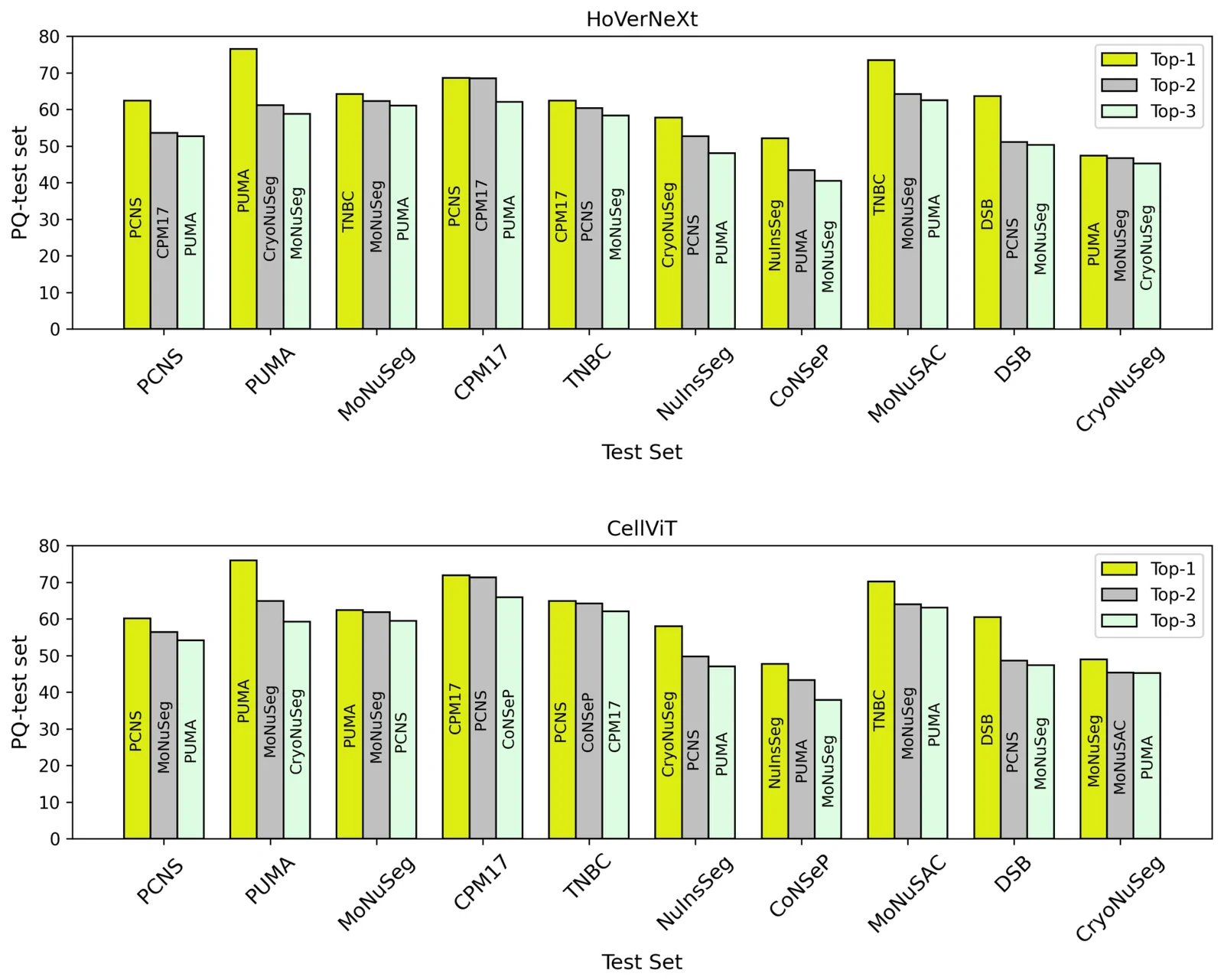
Top three performing training datasets for each test subset for HoVerNeXt (**top**) and CellViT (**bottom**).

We also report the dataset-based cross-dataset score results in Figure 4. In each matrix, the horizontal axis represents the training dataset, while the vertical axis represents the test subset. For clearer visualization, the top three training datasets for each test subset are highlighted in Figure 5.

From Figure 4, we can observe that some values in the correlation matrices exceed 1.0. This occurs when the highest performance on a given test subset was not achieved by training on the corresponding dataset. This means training on a different dataset yielded better performance than training on the dataset’s own training split. As shown in Figure 5, for example, for the HoVerNeXt model, the best performance on the PCNS test subset was achieved when the model was trained on the PCNS training data, which is expected. While this behavior was observed in many cases, there were also notable exceptions. For instance, on the TNBC test subset, the highest performance was obtained when the model was trained on the CPM17 training data rather than on TNBC training data. In this example, TNBC training data was not even among the top three training choices (the second and third choices were PCNS and MoNuSeg training datasets, respectively). Of note, the largest difference between the corresponding upper- and lower-triangular values was observed for the NuInsSeg–CryoNuSeg dataset pair, with a difference of 0.33 for both models. For CellViT, CryoNuSeg showed the largest mean difference between the upper and lower triangles (0.13), followed by MoNuSAC (0.12). For HoVerNeXt, CryoNuSeg again showed the largest mean difference (0.25), followed by TNBC (0.11).

Considering all values across both models, the highest cross-dataset score value is 1.08 (trained on PUMA and tested on CryoNuSeg), and the lowest cross-dataset score value is 0.60 (trained on NuInsSeg and tested on CoNSeP). A cross-dataset score threshold can be defined to determine which datasets can be selected for training. For example, if 0.87 is chosen as the cross-dataset score threshold, then for both models, the PCNS dataset alone is sufficient for training, as it achieves a cross-dataset score above 0.87 across all test subsets.

To further investigate the validity of our reported ranking, we performed an additional experiment using the same training datasets, but testing on the entire PanNuke dataset instead of the NucFuse-test set. The detailed results and rankings based on PQ performance are reported in Table S3 in the Supplementary Materials. As shown in the results, the ranking on the external PanNuke dataset is very similar to that observed on NucFuse-test. Specifically, the top two ranked datasets, PCNS and PUMA, remained unchanged, while MoNuSeg and CPM17 remained among the top four, although their order was switched.

### 4.2. Experiment 2 Results: K-Best Dataset Training

**Figure 6:**
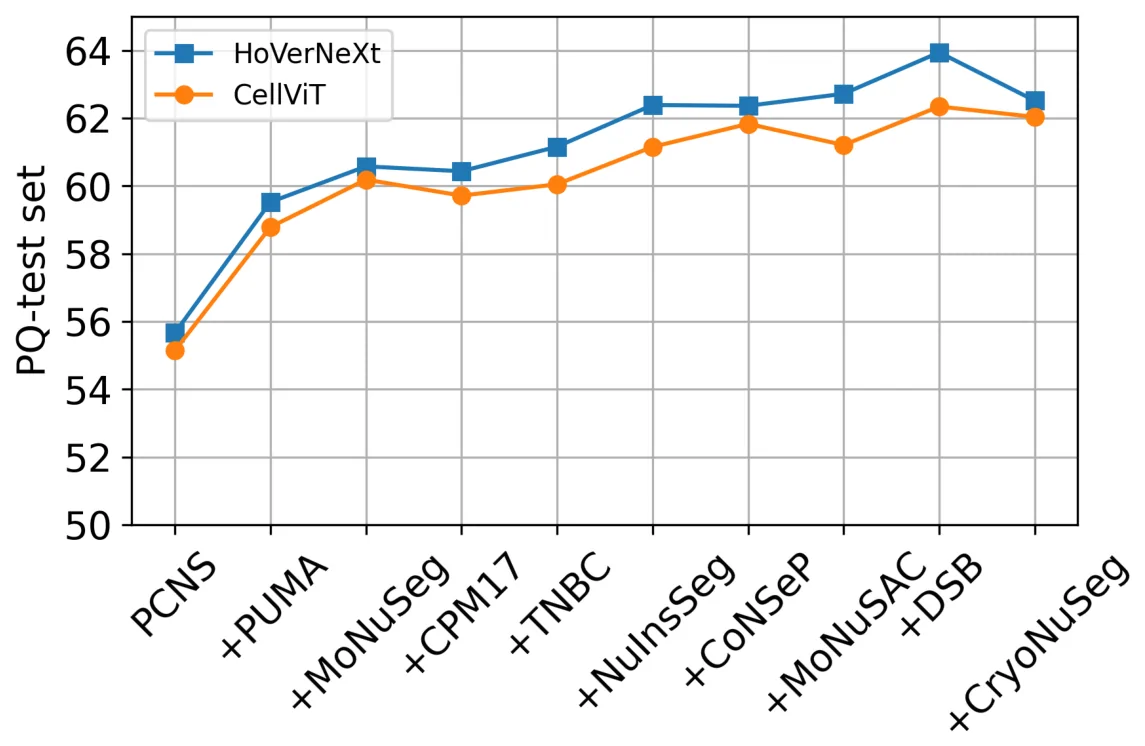
Results of merging the top-*K* best datasets and training the models, evaluated on the NucFuse-test dataset based on panoptic quality (PQ) score (%).

The results of Experiment 2, described in Section 3.2, are shown in Figure 6. For each model, the segmentation performance on the NucFuse-test set, in terms of PQ, is reported after adding each new training dataset. The experiment included models trained with k=1 (only the PCNS training data, which achieved the top overall rank in Experiment 1) up to k=10, which represents training on all datasets. As can be seen for both models, performance increased with each newly added dataset up to k=9. Adding the last dataset, CryoNuSeg, did not lead to improved performance. Therefore, to form the NucFuse-train dataset, we excluded CryoNuSeg and combined the training data from the remaining nine datasets (*k* = 9). As mentioned in Section 4, CryoNuSeg was created from frozen-sectioned samples, which differ substantially from the other datasets that are mainly based on FFPE samples. This domain shift may explain why adding CryoNuSeg to the training data is not beneficial. Nevertheless, we retained CryoNuSeg in the overall analysis, as its inclusion demonstrates that simply adding more datasets to the merged training set does not necessarily lead to improved performance, and that careful dataset selection remains essential. Comparing k=1 and k=9 shows an 8.28% improvement in PQ for HoVerNeXt (from 55.66% to 63.94%) and a 7.20% improvement for CellViT (from 55.14% to 62.34%).

Another outcome to emphasize (Figure 6) is that the largest improvement in performance was achieved when the PCNS, PUMA and MoNuSeg training datasets were merged. While adding additional datasets further increased performance, their effect was less pronounced compared to the performance gain obtained by merging the first two datasets. With the same merging strategy reported in Figure 6, but using PanNuke for testing instead of NucFuse-test, we also observed performance improvements when merging the top three ranked datasets: from 56.37 to 56.97 for HoVerNeXt and from 56.80 to 57.32 for CellViT. Adding the remaining seven datasets led to scores of 57.10 for HoVerNeXt and 56.70 for CellViT, indicating only minor changes in performance. These results further support our main ranking and merging strategy, especially the importance of the top-ranked datasets. However, the overall performance changes were less evident when testing on PanNuke compared with NucFuse-test.

**Figure 7:**
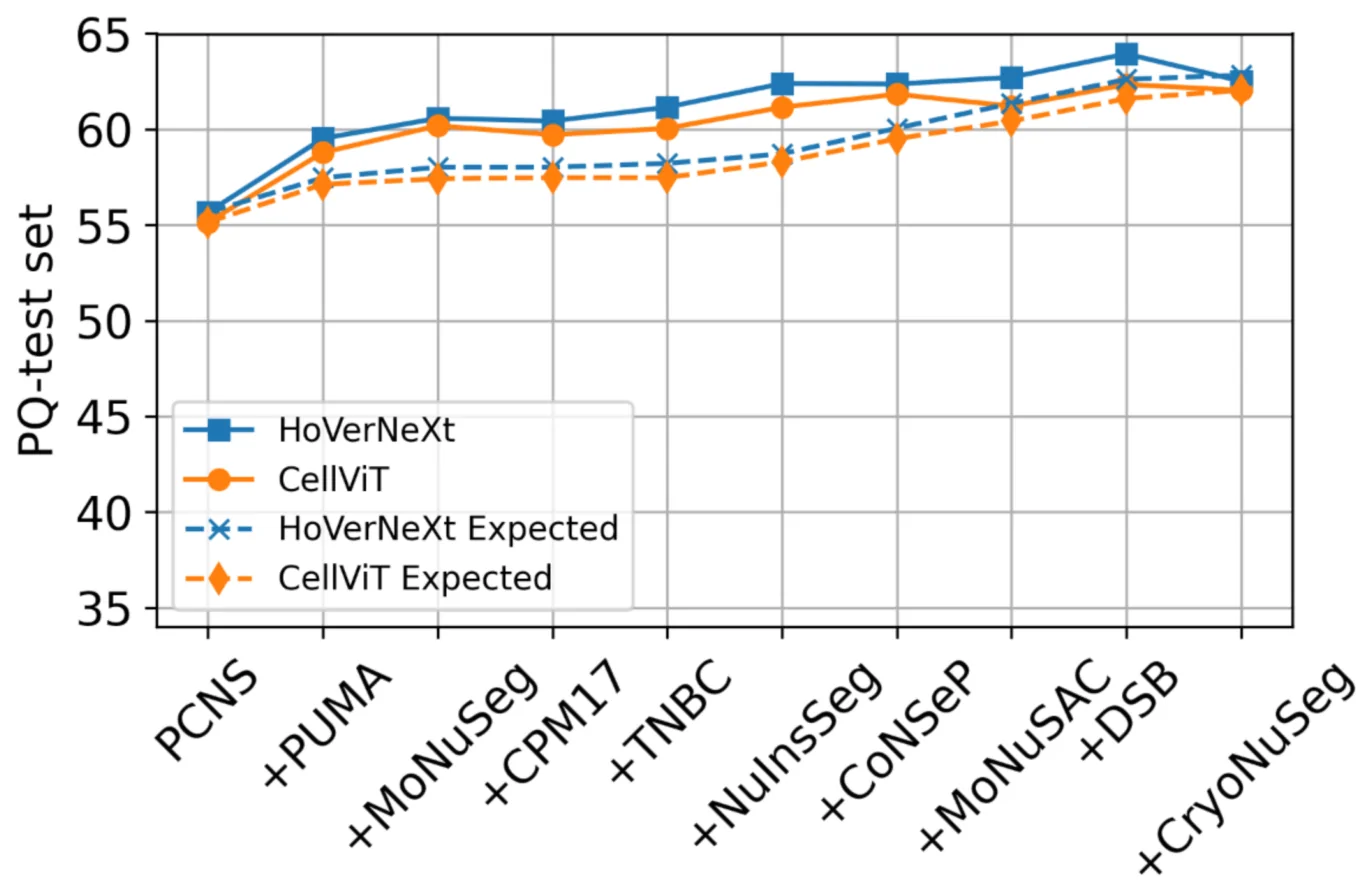
Comparison of the expected dataset fusion improvement with the observed results in Experiment 2.

**Figure 8:**
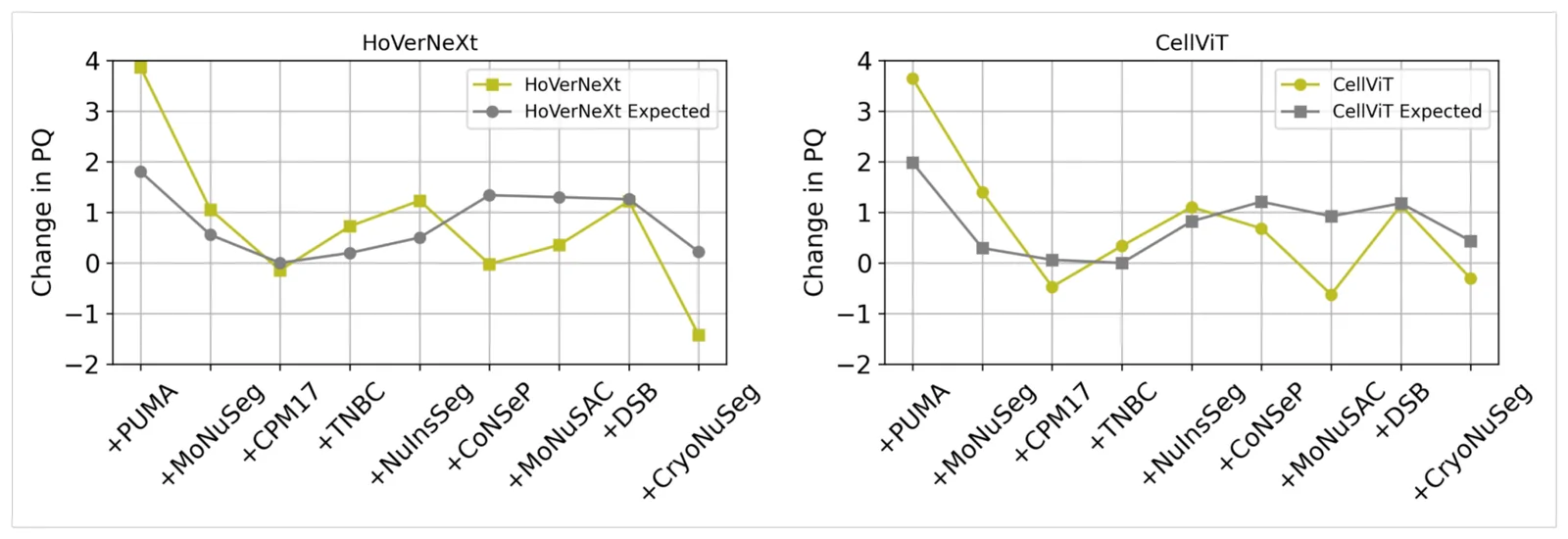
The performance increments for both expected values and observed results for HoVerNeXt and CellViT models.

**Figure 9:**
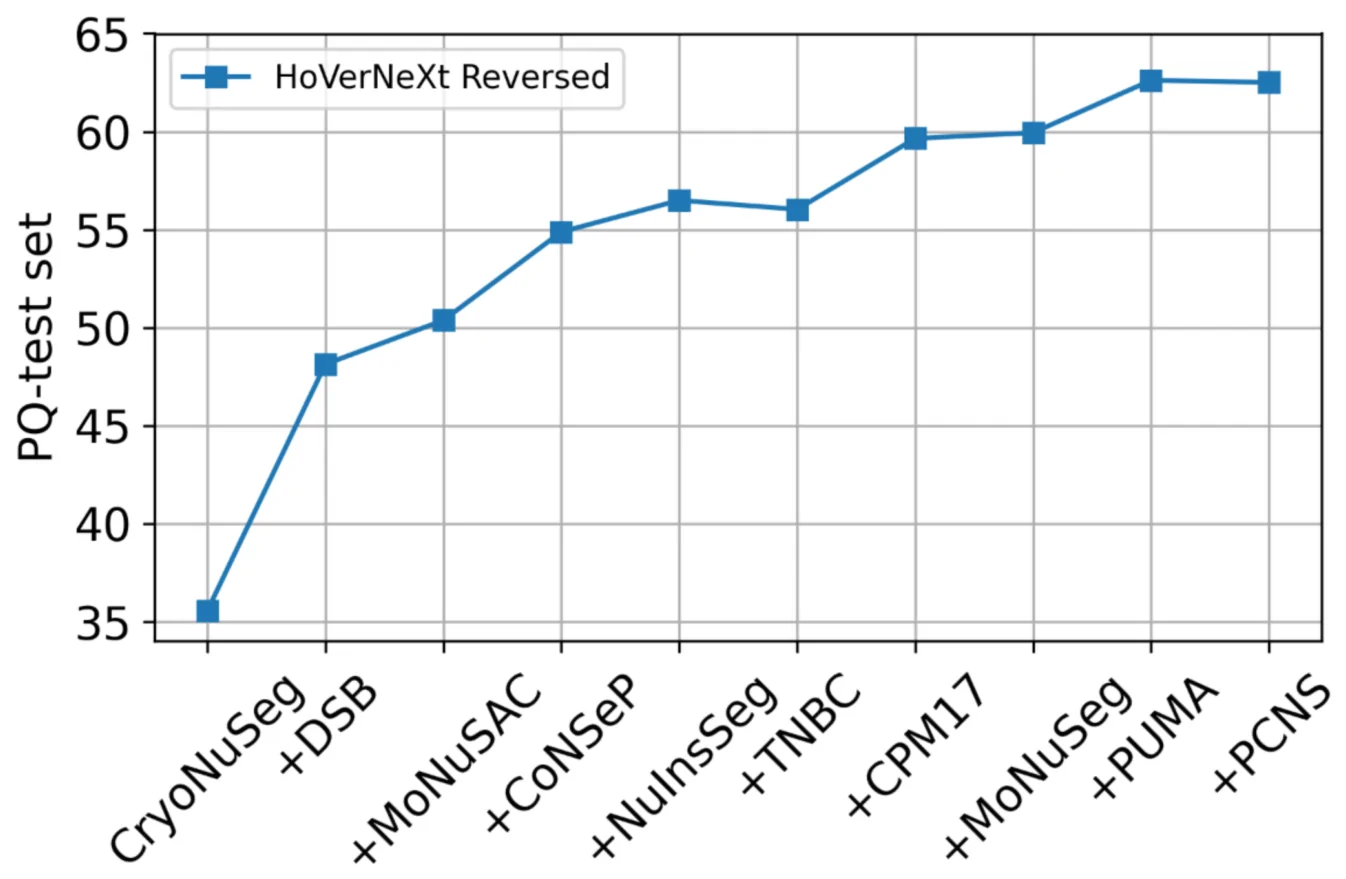
Results of merging the top-K best datasets in reverse order for the NucFuse-test dataset using the HoVerNeXt model.

To better understand the effect of dataset addition, we compared the expected dataset fusion improvement with the results of Experiment 2. The expected fusion improvement represents the maximum performance gain achievable by adding a new dataset for training. For each of the top-*k* datasets, the expected value was calculated using the maximum PQ values from the results of Experiment 1 for the corresponding *k* datasets. Figure 7 presents both the expected and observed results from Experiment 2. As shown, the actual performance gain exceeds the expected improvement. To identify which datasets contribute most to the performance gain, we computed the performance increment for both the expected values and the observed results from Experiment 2, as shown in Figure 8. The figure reveals that some datasets produce a larger performance increase than expected (e.g., PCNS), while others contribute negatively (e.g., CryoNuSeg). A larger improvement in the observed results relative to the expected values suggests that these datasets not only enhance performance on their own samples but also positively contribute to performance on other datasets, indicating a complementary effect. We also performed an additional experiment in which we evaluated the effect of adding the datasets in reverse order for the HoVerNeXt model. The results in Figure 9 demonstrate that adding the datasets in reverse order leads to poorer performance. For example, merging PCNS (rank 1) and PUMA (rank 2) leads to substantially better performance compared to merging CryoNuSeg (rank 10) and DSB (rank 9). These results confirm that higher-ranked datasets contribute more substantially to overall model performance, and that superior segmentation results can be achieved by prioritizing the inclusion of higher-ranked datasets.

### 4.3. Additional Experiments Results

#### 4.3.1. Results from Stain Normalization

**Figure 10:**
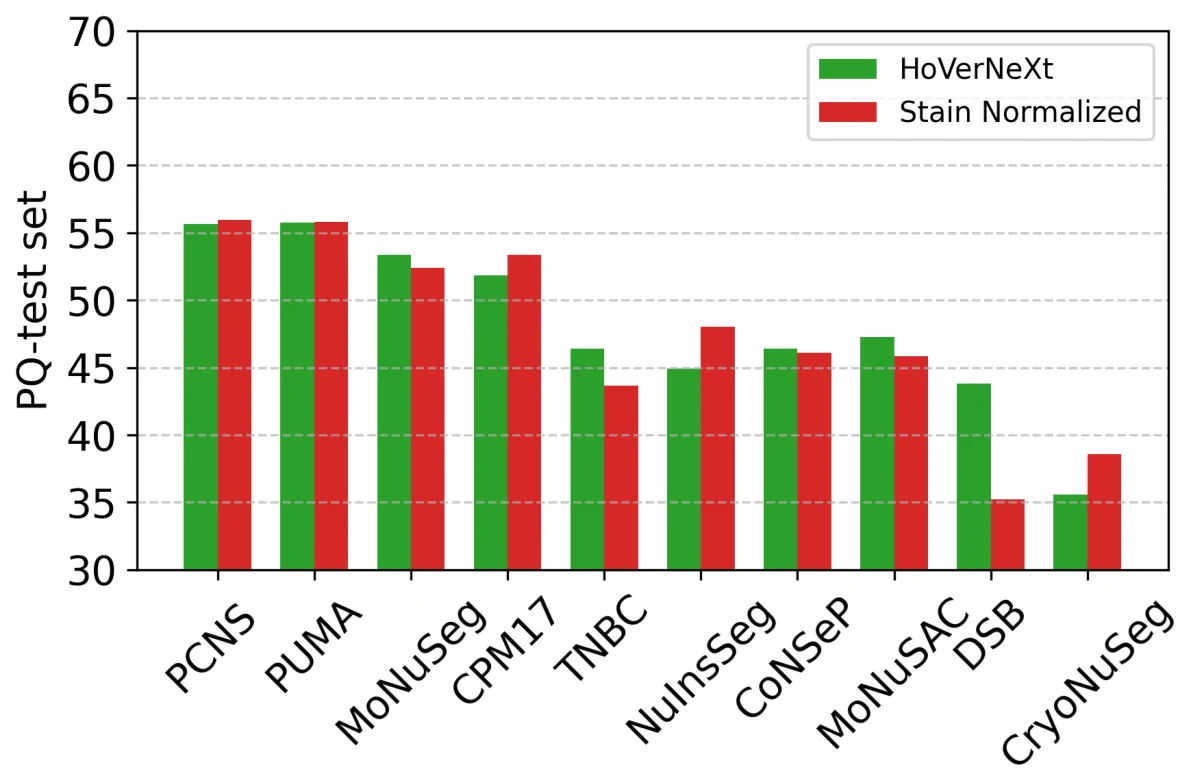
The effect of stain normalization on the performance based on panoptic quality (PQ) score (%). For evaluation, the entire NucFuse-test data was used.

The results of these experiments using the HoVerNeXt model are shown in Figure 10. As can be seen, the effect varies across datasets. Overall, the average performance across all datasets decreased from 48.10% to 47.49%. Among the datasets, CPM17, NuInsSeg, and CryoNuSeg exhibited improved performance, whereas for MoNuSeg, TNBC, MoNuSAC, and DSB, performance decreased when stain normalization was applied. The most substantial decrease was observed for DSB. For PCNS, PUMA, and CoNSeP, performance remained comparable with and without stain normalization. These results confirm previous findings that stain normalization does not always improve performance in histological image analysis tasks [53].

#### 4.3.2. Comparison with PanNuke and CellSAM

The results of these experiments, together with comparisons against HoVerNeXt and CellViT trained on NucFuse-train, are reported in Table 3. As the results indicate, the models trained on PanNuke yielded inferior performance compared to those trained on NucFuse-train. This was observed despite the fact that datasets such as MoNuSeg and CPM17 were used during the creation of PanNuke, which could potentially introduce data leakage. Nevertheless, training on NucFuse-train still yielded superior performance for both HoVerNeXt and CellViT. Compared with training on individual datasets (reported in Table 2), PanNuke delivered superior performance; however, due to potential data leakage from MoNuSeg and CPM17, a direct comparison is not feasible. Compared with CellSAM, training on NucFuse-train achieved higher performance with a large margin. While CellSAM yielded inferior results compared to datasets in group 1 and group 2 in Table 2, it outperformed some datasets in group 3 and all datasets in group 4.

#### 4.3.3. Example Results

**Figure 11:**
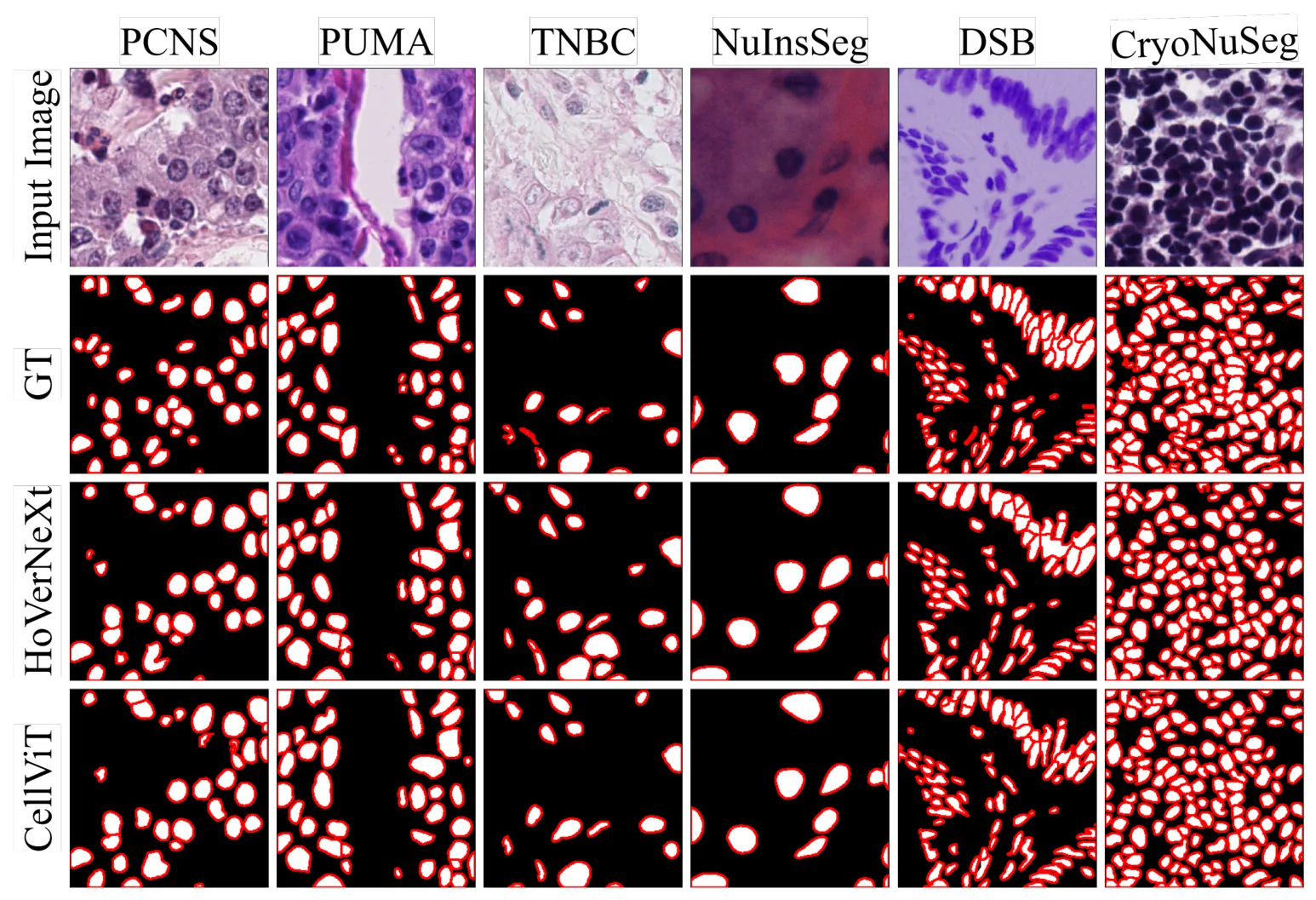
Visualization of nuclei instance segmentation results from HoVerNeXt [21] and CellViT [24]. Both models were trained on NucFuse-train, and their predictions are compared with the ground truth (GT). For each image, a 256×256 pixel crop is shown for better visualization. Input image: H&E-stained images. Color code: white for nuclei, red for nuclei boundaries, and black as background.

Visualization of representative sample results is shown in Figure 11. The input images were randomly selected from the NucFuse-test set from different datasets, and the predictions show the outputs of HoVerNeXt and CellViT when trained on the NucFuse-train dataset.

### 4.4. Limitations

Variations in dataset characteristics, such as the number of patches, patch size, number of organs, and staining variations, make it difficult to compare datasets. In our experiments, we treated these characteristics as intrinsic properties of the datasets and designed a straightforward and unified pipeline based on this assumption.

While our approach enables fair benchmarking, improved results might be achieved for individual datasets through additional preprocessing, alternative sampling strategies during training, or by choosing a larger encoder for the models. For example, applying stain normalization improved performance on NuInsSeg, moving it from eighth to fifth place for HoVerNeXt in Table 2. Another example is CryoNuSeg. CryoNuSeg (the smallest dataset in our experiments) included only 30 image patches in total, of which 14 were used in the unified test set; therefore, only 16 images remained for training, and alternative sampling strategies could yield better results. However, we treated all datasets in a unified manner to enable fair comparison and to identify an optimal joint training strategy. Dataset-specific preprocessing and modifications were therefore not the main focus of this study.

Additionally, in this study, we focused on the nuclei instance segmentation task, whereas nuclei instance segmentation combined with classification could also benefit from a similar investigation. However, considering the different nuclei classes defined across datasets, merging datasets and performing fair comparisons for the joint segmentation-and-classification task becomes more challenging. Nevertheless, this direction is valuable and remains open for future research.

## 5. Conclusions

Dataset quality plays a critical role in training DL models, particularly for histological images characterized by complex tissue anatomy. Over the years, different research groups have introduced several publicly available datasets for nuclei instance segmentation, each with distinct properties. A key open question is determining which of these datasets exhibit greater generalizability and are therefore more suitable for benchmarking nuclei instance segmentation models. To address this, we evaluated the performance of two state-of-the-art models across multiple experimental settings and systematically compared the datasets. Based on our findings, we grouped the datasets into four categories and identified those with the highest potential for the nuclei instance segmentation task. Our results also demonstrate that progressively merging datasets improves segmentation performance in most cases; however, this improvement is not guaranteed, as adding CryoNuSeg did not lead to better segmentation performance. This underscores the importance of careful dataset curation and selection when constructing merged training sets. Additionally, we proposed a unified training set and a unified test set that can serve as a benchmark for future research in nuclei instance segmentation.

## Figures and Tables

**Table 1:** Summary of the datasets used in this study. Datasets listed in the table (No. 1 to No. 10) are manually annotated for the task of nuclei instance segmentation. The PanNuke dataset (No. 11) is a semi-automatically generated dataset, and NucFuse-train and NucFuse-test are merged datasets. PS: patch size (in pixels); CL: classification label; cross marks (×) indicate dataset originally designed for nuclei instance segmentation and check marks (✓) indicate dataset designed for nuclei instance segmentation and classification; ND: average nuclei density in image patches with 256×256 pixels; TP: number of training image patches with 256×256 pixels, with one exception for NucFuse-test, where this value indicates the total number of test images with varying sizes, na: not available.

No.	Name	Patches	Nuclei	Organs	PS	CL	ND	TP
1	PCNS [30]	1356	37,313	9	256 × 256	×	27.51±17.69	1342
2	PUMA [31]	205	97,187	8	1024 × 1024	✓	29.63±9.97	3056
3	MoNuSeg [11]	51	30,827	9	1000 × 1000	×	39.61±24.41	592
4	CPM17 [32]	64	7570	4	500 × 500–600 × 600	×	27.25±13.71	285
5	TNBC [33]	50	4056	1	512 × 512	×	20.28±14.56	144
6	NuInsSeg [25]	665	30,698	31	512 × 512	×	11.54±10.89	2604
7	CoNSeP [20]	41	24,332	1	1000 × 1000	✓	38.89±28.33	432
8	MoNuSAC [29]	310	46,828	4	33 × 86–1771 × 1760	✓	21.31±24.70	2692
9	DSB [34]	107	4529	na	256 × 320	×	33.86±24.68	186
10	CryoNuSeg [28]	30	7592	10	512 × 512	×	63.26±37.66	64
11	PanNuke [35]	7904	189,744	19	256 × 256	✓	24.00±20.12	6320
12	NucFuse-train	2739	248,815	na	33 × 86–1771 × 1760	×	24.25±18.88	11,333
13	NucFuse-test	140	34,525	na	256 × 256–1400 × 1872	×	32.27±25.60	140

**Table 2:** Ranking of datasets based on their mean panoptic quality (PQ) performance (%) on the NucFuse-test set. Precision, recall, and PQ are reported as the mean with the corresponding 95% bootstrap confidence interval in brackets.

Dataset	Model	Precision [95% CI]	Recall [95% CI]	PQ [95% CI]	PQ Rank	Mean Rank
PCNS [30]	HoVerNeXt	72.64 [70.03–75.11]	76.82 [73.76–79.83]	55.66 [53.48–57.89]	2	1
	CellViT	73.21 [70.66–75.73]	74.67 [71.29–78.02]	55.14 [52.78–57.60]	1	
PUMA [31]	HoVerNeXt	78.62 [76.07–80.96]	70.83 [67.34–74.18]	55.75 [53.21–58.22]	1	2
	CellViT	77.89 [75.44–80.19]	69.62 [65.92–73.15]	54.74 [52.07–57.37]	3	
MoNuSeg [11]	HoVerNeXt	71.05 [68.37–73.59]	73.72 [70.49–76.79]	53.38 [51.26–55.45]	3	3
	CellViT	75.09 [72.53–77.50]	72.26 [68.77–75.59]	55.11 [52.65–57.42]	2	
CPM17 [32]	HoVerNeXt	68.05 [65.06–70.98]	71.81 [68.13–75.42]	51.86 [49.24–54.49]	4	4
	CellViT	70.36 [67.33–73.35]	70.01 [66.35–73.60]	51.58 [48.92–54.27]	4	
TNBC [33]	HoVerNeXt	65.82 [62.88–68.73]	62.44 [58.31–66.53]	46.39 [43.74–48.98]	7	5
	CellViT	65.13 [62.11–68.08]	68.85 [65.01–72.58]	48.67 [46.09–51.27]	5	
NuInsSeg [25]	HoVerNeXt	77.01 [73.85–79.97]	55.66 [50.55–60.52]	44.92 [41.24–48.41]	8	6
	CellViT	71.77 [69.00–74.51]	63.35 [59.17–67.32]	48.54 [45.83–51.20]	6	
CoNSeP [20]	HoVerNeXt	61.02 [58.09–63.91]	68.24 [64.62–71.66]	46.41 [44.06–48.74]	6	7
	CellViT	57.05 [54.08–60.01]	67.51 [63.69–71.13]	44.97 [42.43–47.49]	7	
MoNuSAC [29]	HoVerNeXt	77.70 [74.12–81.02]	55.34 [50.87–59.74]	47.28 [43.87–50.62]	5	8
	CellViT	71.44 [67.24–75.39]	45.84 [40.84–50.77]	40.40 [36.45–44.34]	9	
DSB [34]	HoVerNeXt	60.57 [57.24–63.78]	61.97 [57.97–65.87]	43.83 [40.99–46.57]	9	9
	CellViT	49.26 [45.45–53.00]	59.72 [56.16–63.26]	38.44 [35.46–41.32]	10	
CryoNuSeg [28]	HoVerNeXt	60.31 [56.58–63.95]	48.16 [44.08–52.27]	35.57 [32.97–38.10]	10	10
	CellViT	55.90 [51.97–59.62]	59.91 [55.70–64.19]	40.82 [37.77–43.93]	8	
Mean	HoVerNeXt	69.27	64.49	48.10		
(across datasets)	CellViT	66.71	65.17	47.84		

**Table 3:** Performance of HoVerNeXt and CellViT on NucFuse-test and its constituent datasets when trained on either NucFuse-train or PanNuke based on panoptic quality (PQ) score (%). The final column reports CellSAM performance evaluated on the same test sets.

Dataset	HoVerNeXt (NucFuse-train)	CellViT (NucFuse-train)	HoVerNeXt (PanNuke)	CellViT (PanNuke)	CellSAM
PCNS	61.74	60.05	62.10	59.16	40.90
PUMA	77.09	75.24	68.58	67.25	49.88
MoNuSeg	66.61	65.27	65.59	67.24	59.68
CPM17	73.04	73.46	71.73	72.13	62.56
TNBC	66.83	62.02	62.41	66.22	59.75
NuInsSeg	59.72	59.12	48.66	51.54	46.88
CoNSeP	50.54	48.64	50.87	52.34	30.00
MoNuSAC	72.88	70.31	63.81	66.46	60.17
DSB	59.00	60.37	56.94	38.13	42.31
CryoNuSeg	51.95	48.95	50.71	51.26	35.76
**NucFuse-test**	**63.94**	**62.34**	**60.14**	**59.17**	**48.79**

## Data Availability

The datasets used in this study are publicly available and were released with the corresponding previously published papers. The code developed for this study is available on GitHub, with the corresponding links provided in the manuscript.

## Supplementary Materials

The following supporting information can be downloaded at https://www.mdpi.com/article/10.3390/bioengineering13080886/s1. Table S1: Excluded datasets and justification for their exclusion; Table S2: The average panoptic quality (PQ) performance (%) for each test subset of NucFusetest; Table S3: Ranking of datasets based on their mean panoptic quality (PQ) performance on the PanNuke dataset. The refs. [54,55,56,57,58,59,60,61] are citied in Supplementary Materials.

## Abbreviations

The following abbreviations are used in this manuscript:

AI	Artificial Intelligence
CL	Classification Label
CNN	Convolutional Neural Network
DL	Deep Learning
FFPE	Formalin-Fixed Paraffin-Embedded
FN	False Negative
FP	False Positive
GT	Ground Truth
H&E	Hematoxylin and Eosin
IoU	Intersection over Union
na	Not Available
ND	Nuclei Density
PQ	Panoptic Quality
TP	True Positive
PS	Patch Size
TP	Training Patches
ViT	Vision Transformer
WSI	Whole-Slide Image
