# Supplementary material for: NucFuseRank: Dataset Fusion and Performance Ranking for Nuclei Instance Segmentation
Source: Bioengineering (Basel). 2026 Jul 31;13(8):886. doi: 10.3390/bioengineering13080886 (PMC13510264; doi:10.3390/bioengineering13080886)
Supplement: Supplementary file 1 [file bioengineering-13-00886-s001.zip › bioengineering-4422939-supplementary.pdf]

NucFuseRank: Dataset Fusion and Performance Ranking for Nuclei Instance Segmentation

Supplementary Materials

Table S1: Excluded datasets and justification for their exclusion. (Related to Section 2.1.11.)

Dataset	Exclusion reason
Kumar et al. [1]	Extended version available
CPM-15 [2]	Extended version available
OCELOT [3]	Dataset limited to detection task
Post-NAT-BRCA [4]	Dataset limited to detection task
Adipocyte [5]	Dataset limited to detection task
Crowdsource [6]	Dataset includes bounding box annotations
Lizard [7]	Semi-automatically annotated
NuCLS [8]	Semi-automatically annotated
SegPath [9]	Semi-automatically annotated
HistoTRAIN [10]	Semi-automatically annotated
Janowczyk et al. [11]	Weakly annotated dataset
BreCaHAD [12]	Dataset limited to detection task
CRCHisto [13]	Dataset is no longer available
UCSB Bio-Segmentation [14]	Dataset is no longer available

Table S2: The average panoptic quality (PQ) performance (%) for each test subset of NucFuse-test. The best and worst performances are highlighted in bold and underlined, respectively. (Related to Section 4.1.)

**Test Subset**	**HoVerNeXt**	**CellViT**	**Average**
PCNS [15]	46.91	48.32	47.62
PUMA [16]	**57.23**	57.45	57.34
MoNuSeg [17]	55.49	53.87	54.68
CPM17 [2]	56.91	**59.21**	**58.06**
TNBC [18]	46.25	49.84	48.04
NuInsSeg [19]	39.00	37.87	38.44
CoNSeP [20]	<u>33.14</u>	<u>32.64</u>	<u>32.89</u>
MoNuSAC [21]	58.61	58.52	54.68
DSB [22]	44.96	39.65	42.30
CryoNuSeg [23]	42.49	41.01	41.75

Table S3: Ranking of datasets based on their mean panoptic quality (PQ) performance on the PanNuke dataset. Results are reported as percentages, with 95% bootstrap confidence intervals in brackets. (Related to Section 4.1.)

Dataset	Model	PQ (95% CI)	PQ Rank	Mean Rank
PCNS [15	HoVerNeXt	52.98 [52.60–53.37]	2	1
	CellViT	55.91 [55.51–56.30]	1	
PUMA [16]	HoVerNeXt	53.54 [53.12–53.97]	1	2
	CellViT	52.94 [52.52–53.36]	2	
CPM17 [2]	HoVerNeXt	50.53 [50.13–50.93]	3	3
	CellViT	52.88 [52.47–53.28]	3	
MoNuSeg [17]	HoVerNeXt	47.83 [47.45–48.21]	4	4
	CellViT	52 [51.61–52.39]	4	
TNBC [18]	HoVerNeXt	44.95 [44.57–45.32]	5	5
	CellViT	48.92 [48.52–49.31]	5	
CoNSeP [20]	HoVerNeXt	40.52 [40.15–40.90]	7	6
	CellViT	42.37 [41.97–42.77]	7	
NuInsSeg [19]	HoVerNeXt	38.93 [38.44–39.41]	8	7
	CellViT	44.71 [44.28–45.14]	6	
MoNuSAC [21]	HoVerNeXt	41.64 [41.17–42.10]	6	8
	CellViT	41.18 [40.67–41.71]	8	
CryoNuSeg [23]	HoVerNeXt	32.5 [32.11–32.87]	10	9
	CellViT	38.6 [38.19–39.02]	9	
DSB [22]	HoVerNeXt	36.73 [36.35–37.11]	9	10
	CellViT	33.75 [33.36–34.14]	10	
Mean	HoVerNeXt	44.02		
(acroiss datasets)	CellViT	46.32		

References

1. N. Kumar, R. Verma, S. Sharma, S. Bhargava, A. Vahadane, A. Sethi, A dataset and a technique for generalized nuclear segmentation for compu-tational pathology, IEEE Transactions on Medical Imaging 36 (7) (2017) 1550–1560. https://doi.org/10.1109/TMI.2017.2677499.
2. Q. D. Vu, S. Graham, T. Kurc, M. N. N. To, M. Shaban, T. Qaiser, N. A. Koohbanani, S. A. Khurram, J. Kalpathy-Cramer, T. Zhao, R. Gupta,J. T. Kwak, N. Rajpoot, J. Saltz, K. Farahani, Methods for segmenta-tion and classification of digital microscopy tissue images, Frontiers in Bioengineering and Biotechnology Volume 7 - 2019 (2019). doi:https://doi.org/10.3389/fbioe.2019.00053.
3. J. Ryu, A. V. Puche, J. Shin, S. Park, B. Brattoli, J. Lee, W. Jung, S. I. Cho, K. Paeng, C.-Y. Ock, D. Yoo, S. Pereira, Ocelot: Overlapped cell on tissue dataset for histopathology, in: IEEE/CVF Conference on Computer Vision and Pattern Recognition, 2023, pp. 23902–23912. doi:https://doi.org/10.1109/CVPR52729.2023.02289.
4. M. Peikari, S. Salama, S. Nofech-Mozes, A. L. Martel, Automatic cellu-larity assessment from post-treated breast surgical specimens, Cytometry Part A 91 (11) (2017) 1078–1087. doi:https://doi.org/10.1002/cyto. a.23244.
5. J. P. Cohen, G. Boucher, C. A. Glastonbury, H. Z. Lo, Y. Bengio, Count-ception: Counting by fully convolutional redundant counting, in: 2017 IEEE International Conference on Computer Vision Workshops (ICCVW), 2017, pp. 18–26. oi:https://doi.org/10.1109/ICCVW.2017.9.
6. H. Irshad, L. Montaser-Kouhsari, G. Waltz, O. Bucur, J. Nowak, F. Dong,N. W. Knoblauch, A. H. Beck, Crowdsourcing image annotation for nu-cleus detection and segmentation in computational pathology: evaluating experts, automated methods, and the crowd, in: Pacific symposium on biocomputing Co-chairs, 2014, pp. 294–305. doi:https://doi.org/10. 1142/9789814644730_0029.
7. S. Graham, M. Jahanifar, A. Azam, M. Nimir, Y.-W. Tsang, K. Dodd,E. Hero, H. Sahota, A. Tank, K. Benes, N. Wahab, F. Minhas, S. E. A. Raza, H. El Daly, K. Gopalakrishnan, D. Snead, N. Rajpoot, Lizard: A large-scale dataset for colonic nuclear instance segmentation and classifica-tion, in: IEEE/CVF International Conference on Computer Vision Work-shops, 2021, pp. 684–693. doi:https://doi.org/10.1109/ICCVW54120.2021.00082.
8. M. Amgad, L. A. Atteya, H. Hussein, K. H. Mohammed, E. Hafiz, M. A. T. Elsebaie, A. M. Alhusseiny, M. A. AlMoslemany, A. M. Elmatboly, P. A. Pappalardo, et al, Nucls: A scalable crowdsourcing approach and dataset for nucleus classification and segmentation in breast cancer, GigaScience 11 (2022) giac037. doi:https://doi.org/10.1093/gigascience/giac037.
9. D. Komura, T. Onoyama, K. Shinbo, H. Odaka, M. Hayakawa, M. Ochi,R. R. Herdiantoputri, H. Endo, H. Katoh, T. Ikeda, T. Ushiku, S. Ishikawa, Restaining-based annotation for cancer histology segmentation to overcome annotation-related limitations among pathologists, Patterns 4 (2) (2023) 100688. doi:https://doi.org/10.1016/j.patter.2023.100688.
10. B. Adjadj, P.-A. Bannier, G. Horent, S. Mandela, A. Lyon, K. Schutte,U. Marteau, V. Gaury, L. Dumont, T. Mathieu, et al., Towards comprehen-sive cellular characterisation of h&e slides, arXiv preprint arXiv:2508.09926 (2025). doi:https://doi.org/10.48550/arXiv.2508.09926.
11. A. Janowczyk, A. Madabhushi, Deep learning for digital pathology im-age analysis: A comprehensive tutorial with selected use cases, Journal of Pathology Informatics 7 (1) (2016) 29. doi:https://doi.org/10.4103/2153-3539.186902.

12. A. Aksac, D. J. Demetrick, T. Ozyer, R. Alhajj, BreCaHAD: a dataset for breast cancer histopathological annotation and diagnosis, BMC Research Notes 12 (1) (2019) 82.
13. K. Sirin, S. E. A. Raza, Y.-W. Tsang, D. R. Snead, I. A. Cree, N. M. Rajpoot, Locality sensitive deep learning for detection and classification of nuclei in routine colon cancer histology images., IEEE Transaction on Medical Imaging 35 (5) (2016) 1196–1206. doi:https://doi.org/10.1109/TMI.2016.2525803.
14. E. Drelie Gelasca, J. Byun, B. Obara, B. Manjunath, Evaluation and benchmark for biological image segmentation, in: 2008 15th IEEE Interna-tional Conference on Image Processing, 2008, pp. 1816–1819. doi:https://doi.org/10.1109/ICIP.2008.4712130.
15. L. Hou, R. Gupta, J. S. Van Arnam, Y. Zhang, K. Sivalenka, D. Samaras,T. Kurc, J. H. Saltz, Dataset of segmented nuclei in hematoxylin and eosin stained histopathology images of 10 cancer types, The Cancer Imaging Archive, [Data set] (2019). doi:https://doi.org/10.7937/TCIA.2019. 4A4DKP9U.
16. M. Schuiveling, H. Liu, D. Eek, G. E. Breimer, K. P. Suijkerbuijk,W. A. Blokx, M. Veta, A novel dataset for nuclei and tissue segmentation in melanoma with baseline nuclei segmentation and tissue segmentation benchmarks, GigaScience 14 (2025) giaf011. doi:https://doi.org/10. 1093/gigascience/giaf011.
17. N. Kumar, R. Verma, D. Anand, Y. Zhou, O. F. Onder, E. Tsougenis,H. Chen, P.-A. Heng, J. Li, Z. Hu, Y. et al. , A multi-organ nu-cleus segmentation challenge, IEEE Transactions on Medical Imaging 39 (5) (2020) 1380–1391. doi:https://doi.org/10.1109/TMI.2019.2947628.
18. P. Naylor, M. Laé, F. Reyal, T. Walter, Segmentation of nuclei in histopathology images by deep regression of the distance map, IEEE Transactions on Medical Imaging 38 (2) (2019) 448–459. doi:https://doi.org/10.1109/TMI.2018.2865709.
19. A. Mahbod, C. Polak, K. Feldmann, R. Khan, K. Gelles, G. Dorffner,R. Woitek, S. Hatamikia, I. Ellinger, Nuinsseg: A fully annotated dataset for nuclei instance segmentation in h&e-stained histological im-ages, Scientific Data 11 (1) (2024) 295. doi:https://doi.org/10.1038/ s41597-024-03117-2.
20. S. Graham, Q. D. Vu, S. E. A. Raza, A. Azam, Y. W. Tsang, J. T. Kwak,N. Rajpoot, Hover-Net: Simultaneous segmentation and classification of nuclei in multi-tissue histology images, Medical Image Analysis 58 (2019) 101563. doi:https://doi.org/10.1016/j.media.2019.101563.
21. R. Verma, N. Kumar, A. Patil, N. C. Kurian, S. Rane, S. Graham, Q. D. Vu, M. Zwager, S. E. A. Raza, N. Rajpoot, et al. A. Sethi, Monusac2020: A multi-organ nuclei segmentation and classification challenge, IEEE Transactions on Medical Imaging 40 (12) (2021) 3413–3423. doi:https://doi.org/10.1109/TMI.2021.3085712.
22. J. C. Caicedo, A. Goodman, K. W. Karhohs, B. A. Cimini, J. Ackerman,M. Haghighi, C. Heng, T. Becker, M. Doan, C. McQuin, M. Rohban,S. Singh, A. E. Carpenter, Nucleus segmentation across imaging experi-ments: the 2018 data science bowl, Nature Methods 16 (12) (2019) 1247–1253. doi:https://doi.org/10.1038/s41592-019-0612-7.
23. A. Mahbod, G. Schaefer, B. Bancher, C. Löw, G. Dorffner, R. Ecker, Ellinger, CryoNuSeg: A dataset for nuclei instance segmentation of cryosectioned H&E-stained histological images, Computers in Biology and Medicine 132 (2021) 104349. doi:https://doi.org/10.1016/j. compbiomed.2021.104349.
